# Chemical Composition and Antifungal Activity of *Myrcia multiflora* and *Eugenia florida* Essential Oils

**DOI:** 10.3390/molecules26237259

**Published:** 2021-11-30

**Authors:** Oberdan Oliveira Ferreira, Silvia Helena Marques da Silva, Mozaniel Santana de Oliveira, Eloisa Helena de Aguiar Andrade

**Affiliations:** 1 Programa de Pós-Graduação em Biodiversidade e Biotecnologia-Rede Bionorte, Instituto de Ciências Biológicas, Universidade Federal do Pará, Rua Augusto Corrêa S/N, Guamá, Belém 66075-900, Brazil; oberdan@museu-goeldi.br (O.O.F.); eloisa@museu-goeldi.br (E.H.d.A.A.); 2 Seção de Bacteriologia e Micologia LabMicol—SABMI Laboratório de Micologia, Instituto Evandro Chagas—IEC/SVS/MS, Rodovia BR 316 KM 07, Levilândia, Ananindeua 67030-000, Brazil; silviasilva@iec.gov.br; 3 Laboratório Adolpho Ducke, Coordenação de Botânica, Museu Paraense Emílio Goeldi, Av. Perimetral, 1901, Terra Firme, Belém 66077-830, Brazil

**Keywords:** essential oil, volatile compounds, pathogens, inhibition potential, antifungal action

## Abstract

The essential oils of three specimens of *Myrcia multiflora* (A, B and C) and *Eugenia florida* were extracted by hydrodistillation, and the chemical compositions from the essential oils were identified by gas chromatography and flame ionization detection (CG/MS and CG-FID). The fungicide potential of the EOs against five fungicide yeasts was assessed: *Candida albicans* INCQS-40175, *C. tropicalis* ATCC 6258, *C. famata* ATCC 62894, *C. krusei* ATCC 13803 and *C. auris* IEC-01. The essential oil of the specimen *Myrcia multiflora* (A) was characterized by the major compounds: α-bulnesene (26.79%), pogostol (21.27%) and δ-amorphene (6.76%). The essential oil of the specimen *M. multiflora* (B) was rich in (*E*)-nerolidol (44.4%), (E)-γ-bisabolene (10.64%) and (*E*,*E*)-α-farnesene (8.19%), while (*E*)-nerolidol (92.21%) was the majority of the specimen *M. multiflora* (C). The sesquiterpenes seline-3,11-dien-6-α-ol (12.93%), eremoligenol (11%) and γ-elemene (10.70%) characterized the chemical profile of the EOs of *E. florida*. The fungal species were sensitive to the essential oil of *M. multiflora* (B) (9–11 mm), and the lowest inhibitory concentration (0.07%) was observed in the essential oil of *M. multiflora* (A) against the yeasts of *C. famata*. Fungicidal action was observed in the essential oils of *M. multiflora* (A) against *C. famata*, with an MIC of 0.78 µL/mL and 3.12 µL/mL; *C. albicans*, with an MFC of 50 µL/mL and *M. multiflora* (C) against *C. albicans*; and *C. krusei*, with a MFC of 50 µL/mL.

## 1. Introduction

Fungi are defined as eukaryotic beings that cause huge economic and ecological impacts to society [1]. One of the reasons is food contamination. In addition, in some cases they can be pathogenic due to the production of mycotoxins [2], which favor the triggering of relatively mild diseases and infections, such as in the skin and mucosa, and can worsen, affecting multiple organs [3], such as nails, lungs and hair [4]. Among the fungi that cause human pathologies, we have the species of the *Candida* genus, which are capable of altering, through infections, the immunological suppression or impairment of epithelial barriers [5].

Another concern related to fungi is related to their possible resistance that can be acquired to some commercial drugs. In addition, synthetic products can cause contamination and change food properties. With this, the search for products of natural origin with antimicrobial activity has been increasing over the years [6]. Among the potential bioactive compounds, we highlight those present in essential oils, which can be viable alternatives for the control of fungi [7,8,9,10].

In this context, we can mention the Myrtaceae family, which holds a vast amount of species that produce essential oils [11] and has great antimicrobial potential already described in several species such as *Eucalyptus camaldulensis* [12], *E. lehmannii* [13], *E. sideroxylon* [13], *E. leucoxylon* [13], *E. camaldulensis* [13], *E. astringens* [13], *Eugenia jambolana* [14], *Callistemon citrinus* [14], *Myrtus communis* [14], *Melaleuca genistifolia* [14] and *M. alternifolia* [14]. However, the antimicrobial potential of other species, such as *Myrcia multiflora* and *Eugenia florida*, is unknown.

*Myrcia multiflora* is described as a shrub-sized species [11,15] and is popularly known as pedra-ume-caá or insulin plants. Due to traditional and empirical knowledge, it is used as a hypoglycemic agent in the form of infusion or decoction [16,17]. On the other hand, *Eugenia florida* is a small tree, approximately 3.5 m high, traditionally known as black cherry, whose fruits are edible and tasty. The leaves of this species are used by rural populations as hypotensive and antipyretic agents to reduce cholesterol levels and treat infections, jaundice, heart disease and gastrointestinal disorders [18]. Given the significant importance of these species, the purpose of this study was to assess the chemical composition and the antimicrobial potential of three specimens of *M. multiflora* and one specimen of *E. florida* collected in the municipality of Magalhães Barata, State of Pará, Brazil.

## 2. Results and Discussions

### 2.1. Yield of the Essential Oils

The essential oil of the specimen *Myrcia multiflora* (C) showed the highest yield of 0.98 (*v*/*w* %), followed by the specimen *M. multiflora* (A) with 0.60 (*v*/*w* %), specimen *M. multiflora* (B) with 0.28 (*v*/*w* %) and *Eugenia florida* with 0.13 (*v*/*w* %). These results demonstrate that the collection periods and ecosystems directly impacted the differences in these contents, as well as environmental factors, such as rainfall, solar incidence, temperature and type of soil [19,20,21].

Comparing our study with other works, the yields of the essential oils found in this research for the three specimens of *M. multiflora* were higher than those found by [22] in a sample taken in the municipality of Taquari, State of Rio Grande do Sul, Brazil, with a content of 0.20 (*v*/*w* %), and lower than the sample of *M. multiflora* taken in the municipality of Maracanã, State of Pará, Brazil, with a yield of 1.16 (*v*/*w* %) [23]. The yield of 0.8–3.1% (*v*/*w* %) of the essential oil of *E. florida* was within the average range found in other studies of species of the genus *Eugenia* [24].

### 2.2. Chemical Composition

The essential oils of the studied samples were obtained by hydrodistillation and identified by chromatography coupled to mass spectrometry (GC/MS). Table 1 shows the 100 chemical compounds found in the essential oils of *Myrcia multiflora* (A), *M. multiflora* (B), *M. multiflora*. (C) and *Eugenia florida*, and the total contents of these essential oils ranged from 88.14 to 98.48%. The essential oil of the specimen *Myrcia multiflora* (A) was characterized by the major compounds α-bulnesene (26.79%), pogostol (21.27%) and δ-amorphene (6.76%).

α-bulnesene is described in the literature for presenting antimicrobial activity against fungi and bacteria [25]. In relation to the compound pogostol, studies report its antimicrobial potential [26], and also one of the compounds responsible for the cytotoxic action against cancer cells is present [27]. The antimicrobial, cytotoxic and antioxidant potential of δ-amorphene may be related to its strong presence in research on essential oils [28,29].

The essential oil of the specimen *Myrcia multiflora* (B) was rich in (*E*)-nerolidol (44.4%), (*E*)-γ-bisabolene (10.64%) and (*E*,*E*)-α-farnesene (8.19%) (Table 1), while (E)-nerolidol (92.21%), (*E*,*E*)-α-farnesene (3.28%) and *E*-caryophyllene (1.11%) were the majority for the specimen *Myrcia multiflora* (C). Our results differed from those found in two studies with the essential oil of *Myrcia multiflora*, the first from a sample collected in the municipality of Taquari, State of Rio Grande do Sul, Brazil, which presented β-caryophyllene (7.5%) and germacrene D (8.7%) as the main constituents [22]. The second was from a sample collected in the municipality of Maracanã, State of Pará, Brazil, having as the main compounds β-caryophyllene (10.72%) and selin-11-en-4α-ol (10.67%) [23] (Table 1).

(*E*)-γ-bisabolene is characterized in studies by demonstrating insecticidal activities against mosquitoes [30], and the sesquiterpene (*E*,*E*)-α-farnesene, in the study carried out by [31], as the major constituent demonstrated strong activity antifungal against *Trichophyton rubrum*- and *T. mentagrophytes*-type fungi. Regarding the compound *E-caryophyllene*, studies highlight its fungicidal importance [32] and antioxidant [33], anti-inflammatory [34] and antiprotozoal [35] properties.

The chemical profile of the essential oils of specimen *M. multiflora* was characterized by hydrocarbon and oxygenated sesquiterpenes, and studies on the essential oils of the *Myrcia* genus have proven strong the presence of these classes of compounds, as described in two chemical types presented by the essential oils of two specimens of *M. tomentosa*. Type (A) was characterized by γ-elemene (12.52%), germacrene D (11.45%) and (*E*)-caryophyllene (10.22%), while type (B) was characterized by the major compounds spathulenol (40.70%), α-zingiberene (9.58%) and γ-elemene (6.89%) [36] (Table 1).

There was a difference in the chemical composition between the studied specimens of *M. multiflora*, with specimen (A) differing from the other specimens, but the major compound (*E*)-nerolidol characterized the chemical profile of both specimen (B) and (C). In the latter specimen, the highest content of this sesquiterpene oxygenated was observed (Table 1). This high content was also observed in the essential oil of *M. bracteata*, which presented 80.8% (*E*)-nerolidol [37].

**Table 1:** Chemical composition found in the essential oils of *Myrcia multiflora* and *Eugenia florida*.

IR_L_	IR_C_	Constituents	*M. multiflora* (A)	*M. multiflora* (B)	*M. multiflora* (C)	*E. florida*
1335	1334	δ-elemene	0.26			0.55
1345	1345	α-cubebene	0.13			0.05
1373	1369	α-ylangene	0.06			0.03
1374	1374	α-copaene	0.59	0.03		0.26
1387	1387	β-bourbonene		0.04		0.05
1389	1388	β-elemene	2.1	0.04		1.04
1409	1405	α-gurjunene	0.02			
1411	1412	α-*cis*-bergamotene		0.06		
1417	1418	*E*-caryophyllene	3.66	2.88	1.11	2.35
1430	1428	β-copaene		0.03		
1432	1431	α-*trans*-bergamotene		0.76	0.05	1.78
1434	1432	γ-elemene				10.70
1437	1435	α-guaiene	2.88			
1439	1440	aromadendrene				0.23
1442	1444	6,9-guaiadiene	0.79			
1447	1448	isogermacrene D				0.11
1451	1453	*trans*-muurola-3,5-diene				0.31
1452	1454	α-humulene	3.8			0.39
1454	1455	(*E*)-β-farnesene		4.38	0.59	
1457	1458	β-santalene		0.2		
1458	1459	*allo*-aromadendrene	0.33			0.08
1471	1474	dauca-5,8-diene		0.03		0.64
1478	1479	γ-muurolene		0.12		0.69
1479	1480	amorpha-4,7(11)-diene	3.1			
1481	1481	γ-curcumene		0.18		
1483	1482	β-trans-bergamotene		0.15		
1484	1484	germacrene D	4.12	0.72	0.23	2.21
1489	1488	β-selinene	2.26			0.96
1492	1490	β-cis-guaiene		0.06		
1493	1492	α-zingiberene		1.73		
1493	1495	*trans*-muurola-4(14),5-diene				0.51
1495	1496	γ-amorphene		0.12		
1496	1498	viridiflorene	1.49			1.55
1500	1501	α-muurolene		0.12		0.47
1505	1506	(*E*,*E*)-α-farnesene		8.19	3.28	
1505	1507	β-bisabolene		6.83		
1506	1508	(*Z*)-α-bisabolene		0.2		
1509	1512	α-bulnesene	26.79			
1511	1514	δ-amorphene	6.76	0.54		0.31
1513	1515	γ-cadinene				0.61
1514	1517	(*Z*)-γ-bisabolene				3.41
1515	1518	sesquicineole			0.02	
1521	1522	β-sesquiphellandrene		0.79		
1522	1525	δ-cadinene				4.42
1527	1528	(*E*)-γ-bisabolene		10.64	0.57	
1528	1529	zonarene				0.65
1531	1530	γ-vetivenene				1.59
1532	1531	γ-cuprenene		0.05		
1533	1532	*trans*-cadina-1,4-diene	0.49			0.36
1537	1535	α-cadinene	0.06			
1540	1539	selina-4(15),7(11)-diene				0.99
1540	1542	(*E*)-α-bisabolene		0.25		
1544	1543	α-calacorene	0.07			
1548	1547	elemol	0.07			
1550	1551	*cis*-muurol-5-en-4-α-ol		0.04		
1554	1553	β-vetivenene				4.59
1559	1557	germacrene B	0.08			2.17
1561	1565	(*E*)-nerolidol		44.4	92.21	
1566	1568	maaliol	0.23			
1577	1575	spathulenol	0.52	1.36		1.2
1582	1581	caryophyllene oxide		0.87		
1590	1586	globulol	1.16	0.12		
1592	1592	viridiflorol	1.82			1.23
1596	1595	fokienol		0.18		3.9
1602	1602	ledol	0.17			
1607	1605	5-*epi*-7-*epi*-α-eudesmol	0.29			
1607	1606	(Z)-sesquilavandulol		0.05		
1608	1610	β-atlantol		0.07		0.96
1627	1627	1-*epi*-cubenol	2.39			
1629	1631	eremoligenol	0.31	0.4		11.0
1631	1634	muurola-4,10(14)-dien-1-β-ol				1.24
1632	1635	α-acorenol		0.17		
1635	1636	cis-cadin-4-en-7-ol		0.14		
1636	1637	gossonorol		0.6	0.01	
1640	1639	*epi*-α-muurolol		0.14	0.04	
1642	1641	selina-3,11-dien-6-α-ol				12.93
1644	1645	α-muurolol		0.72		
1645	1646	cubenol	3.17		0.04	2.5
1651	1653	pogostol	21.27			
1652	1654	α-cadinol		0.41		3.98
1658	1658	*neo*-intermedeol			0.09	
1666	1665	14-hydroxy-(*Z*)-caryophyllene		0.35		
1670	1667	bulnesol	0.92			
1670	1670	*epi*-β-bisabolol		0.64		
1674	1674	β-bisabolol			0.09	
1677	1678	mustakone				0.07
1679	1680	khusinol				0.96
1683	1685	*epi*-α-bisabolol		0.46		
1685	1688	α-bisabolol		1.23	0.28	
1685	1688	germacra-4(15),5,10(14)-trien-1-α-ol				0.06
1700	1701	eudesm-7(11)-en-4-ol				1.91
1713	1709	14-hydroxy-α-humulene		0.19		
1728	1733	(*Z*)-γ-curcumen-12-ol		0.26		
1745	1740	γ-costol				0.45
1754	1749	(*Z*)-β-curcumen-12-ol		2.99		
1755	1752	α-sinensal		1.62		
1766	1666	β-costol				0.18
1767	1667	13-hydroxy-valencene				1.51
1777	1778	(*Z*)-α-santalol acetate		1.47		
1794	1799	(*Z*)-α-*trans*-bergamotol acetate		0.46		
		hydrocarbon sesquiterpenes	59.84	39.14	5.83	44.06
		oxygenated sesquiterpenes	32.32	59.34	92.78	44.08
		Total	92.16	98.48	98.61	88.14

IR_C_: calculated from a series of n-alkanes (C8–C40) in a DB-5MS column capillary column, IR_L_: [38,39]; IR_C_: calculated retention index; IR_L_: literature retention index.

The essential oil of *Eugenia florida* was strongly characterized by the sesquiterpenes selina-3,11-dien-6-α-ol (12.93%), eremoligenol (11%) and γ-elemene (10.70%), very different from what was found in a sample taken in Rio Grande do Sul, Brazil, which presented the major compounds bicyclogermacrene (10.9%), germacrene D (10.4%) and β-caryophyllene (8.1%) [40]. Furthermore, the chemical profile of the essential oil of *E. florida* differed from that found in the essential oils of the specimens of *M. multiflora* and that found in the essential oil of *Eugenia* species.

The compounds 5-hydroxy-cis-calemenene (35.8%), β-caryophyllene (8.9%), trans-cadina-1,4-diene (6.3%), trans-calamenene (6.1%), *trans-muurola*-3,5-diene (5.9%) and ledol (5.0%) were the main findings in the essential oil of *E. egensis.* On the other hand, in the essential oil of *E polystachya*, germacrene D (18.4%), ishwarane (15.7%), 7-epi-α-selinene (7.5%) and bicyclogermacrene (5.1%) predominated [41]. The compounds γ-elemene (25.89%), germacrene B (8.11%) and (*E*)-caryophyllene (10.76%) were the main compounds of the essential oil of *E. patrisii* studied by [36], in which, on the other hand, the γ-elemene content was higher than that found in our study. This hydrocarbon sesquiterpene is reported in essential oil research for its antimicrobial and antiproliferative action [42], while the compound seline-3,11-dien-6-α-ol is described in the literature as a possible insecticidal agent [43] and antioxidant [44].

**Figure 1:**
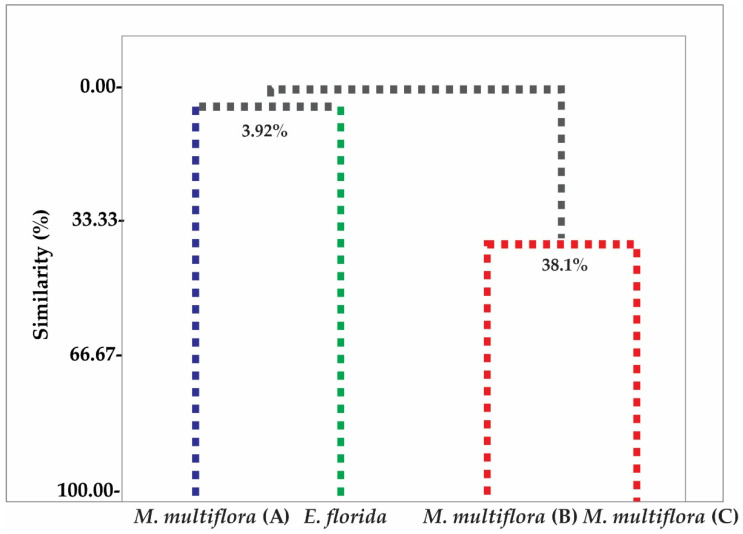
Dendrogram showing relational similarity of compound identified in *M. multiflora* (A), *M. multiflora* (B), *M. multiflora* (C) and *E. florida* essential oil.

**Figure 2:**
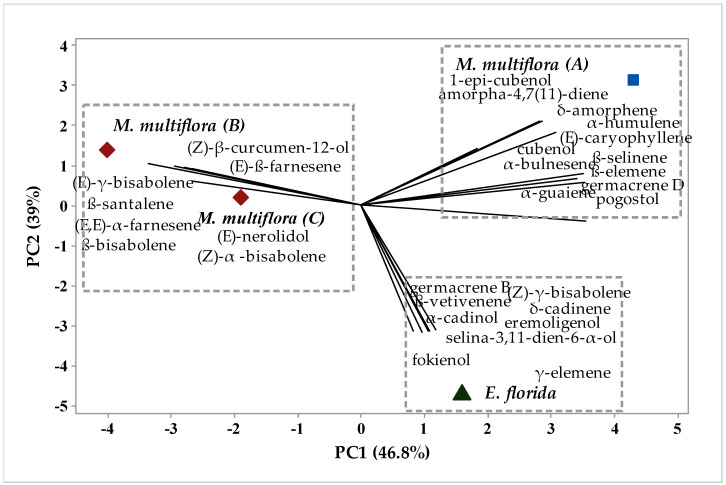
Biplot (PCA) from analysis of compound identified in *M. multiflora* (A) *M. multiflora* (B) *M. multiflora* (C) *E. florida* essential oil.

To analyze the possible similarity in the chemical composition of the samples, chemometric analysis of hierarchical clustering analysis (HCA) and principal component analysis (PCA) were applied to the chemical profiles of the essential oils of *M. multiflora* (A), *M. multiflora* (B), *M. multiflora* (C) and *E. florida*, as shown in Figure 1 and Figure 2, respectively.

With the HCA cluster analysis using Euclidean distance, it was possible to identify the presence of three distinct groups of chemical profiles: Group I, formed by the sample *M. multiflora* (A); Group II, formed by the sample *E. florida*, both with a significant difference of approximately 96.08%; and Group III, formed by the samples *M. multiflora* (B) and *M. multiflora* (C) with a degree of similarity of 38.1% (Figure 1).

In Figure 2, it is possible to confirm the formation of the groups. The compounds responsible for the group formations are presented. The eigenvalues corresponding to the principal component analysis PC1 explained 46.8% of the variance, while PC2 explained 39%. When adding the components, they explain 85.8% of the variation. Moreover, when the groups present in Figure 2 are analyzed, we note the compounds that had the highest weights for their formations. Group I, comprising *M. multiflora* (A), was characterized by the presence of the 1-epi compounds -cubebol, amorpha-4,7(11)-diane, δ-amorphene, α-humulene, (*E*)-caryophyllene, cubeball, β-selinene, α-bulnesene, β-elemene, germacrene D, α-guaiene and pogostol. Group II, comprising *E. florida*, was characterized by the presence of germacrene B, (Z)-δ-bisabolene, β-vetivenene, δ-cadinene, α-candinol, eremoligenol, seline-3,11-dien-6-α-ol, fokienol and γ-elemene. Group III, comprising *M. multiflora* (B) and *M. multiflora* (C), was characterized by the compounds (*Z*)-β-curcumen-12-ol, (*E*)-β-farnesene, (*E*)-*d*-bisabolene, β-santalene, (*E*,*E*)-α-farnesene, β-bisabolene, (*E*)-nerolidol and (*Z*)-α-bisabolene.

### 2.3. Antimicrobial Activity

The in vitro antifungal activity of the four oils was assessed against five fungal species (*Candida albicans* INCQS-40175, *C. tropicalis* ATCC 6258, *C. famata* ATCC 62894, *C. krusei* ATCC 13803 and *C. auris* IEC-01) of medical importance. All tested oils showed an inhibition zone for all assessed species. However, the inhibitory effect varied among the species. The diameter of the halos was between 6 and 11 mm (Table 2).

The studied fungal species showed sensitivity in the presence of the essential oil of the specimen *M. multiflora* (B), which showed higher inhibition power (9–11 mm), based on the size of the halos. These values are consistent with the inhibition potential presented by the antifungal agent amphotericin B against *Candida albicans* (10–19 mm), *C. tropicalis* (10–20 mm) and *C. krusei* (9–20 mm) [45]. The fungal species *C. krusei* showed resistance (6 mm) in contact with the essential oil of the specimen *M. multiflora* (A), in contrast to the other fungal species. The essential oil showed an inhibition potential of 8–9 mm. The species *C. albicans* and *C. krusei* showed resistance to the essential oil of *E. florida*, with inhibition potentials of 7 mm and 6 mm, respectively. The essential oil of the specimen *M. multiflora* (C) showed low inhibition potential (7 mm) against *C. albicans*.

The essential oils from *Myrcia* and *Eugenia* showed high inhibition potential against different types of fungi. The essential oil of *Myrcia ovata* showed significant antimicrobial activity against *C. albicans* (30 mm) [46]. In another study, the essential oil of *E. caryophyllata* was effective against *C. albicans* (18.4 mm) and *C. tropicalis* (19 mm) [47]. These inhibition potentials were higher than those found in our study, which may be somehow related to the chemical type presented in each of the essential oils [48].

By analyzing the minimum inhibitory concentration (MIC), the essential oil of the specimen *M. multiflora* (A) presented 0.78 µL/mL, according to Table 3. This was the lowest MIC found and was able to inhibit the growth of *Candida famata.* This high capacity to inhibit the fungal yeast (*C. famata*) may be related to the main constituents of the essential oil of *M. multiflora* (A): α-bulnesene (26.79%), pogostol (21.27%) and δ-amorphene (6.76%), as well as other compounds with lower essential oil [49]. In turn, the essential oil of the specimen *M. multiflora* (C) showed a minimum inhibitory concentration of 12 µL/mL for both C. krusei and *C. famata.* The MIC values found in this work differed from those observed by [50] in the essential oil of *Siparuna guianensis*, which was able to inhibit the growth of *C. albicans*, with an MIC of 125 µL/mL. Likewise, the essential oil of *Eugenia caryophyllus* was able to inhibit the growth of the same fungal species, with an MIC of 0.017 µL/mL [51].

In this study, it was possible to observe that some essential oils were able to inhibit up to two fungal species. However, others were able to reduce fungal growth, such as the essential oil of the specimen *M. multiflora* (A), which had fungicidal action on *C. albicans* and *C. famata*, with an MFC of 50 µL/mL and 3.12 µL/mL, respectively. On the other hand, the essential oil from the specimen *M. multiflora* (C) had fungicidal action on *C. albicans* and *C. krusei*, with an MFC of 50 µL/mL. It is important to mention that this essential oil is characterized by a high content of (*E*)-nerolidol (92.21%), and this compound is even described in the literature for presenting antimicrobial activity, mainly antifungal [52,53,54]. The MFC result found in the EOs of *M. multiflora* (A) against *C. famata* is within that observed in the essential oil of the leaves of *Myrtus communis* against the fungal species *Candida albicans* (ATCC 10261), *C. tropicalis* (ATCC 750), *C. krusei* (ATCC 6258), *C. glabrata* (ATCC 90030) and *C. parapsilosis* (ATCC 4344), with MFC values ≤8 µL/mL [55].

No report on the antimicrobial activity of the essential oil of *M. multiflora* and *Eugenia florida* was found in the literature. However, studies were observed within the genera *Eugenia* and *Myrcia*, species with antimicrobial properties, of which we can highlight *Eugenia uniflora* [56], *E. involucrata* [57], *E. caryophyllata* [58], *Myrcia palustres* [48], *M. ovata* [59] and *M. splendens* [60].

Other analyses shall be made in order to assess the degree of involvement of the fungal cell better after being challenged with the botanical extracts. Therefore, the antimicrobial properties of the essential oils may be associated with terpene compounds, which, due to their highly lipophilic nature and low molecular weight, are capable of interrupting the cell membrane, causing cell death or inhibiting the sporulation and germination of fungi that deteriorate food products [61].

## 3. Materials and Methods

### 3.1. Botanic Material

Aerial parts of three specimens of *Myrcia multiflora* and one specimen of *Eugenia florida* were collected in the coastal region of the State of Pará, in the city of Magalhães Barata, Brazil. Specimens (A) and (B) of *M. multiflora* were collected on 12/07/2017, the first in an area of secondary forest (capoeira), and the second in areas of swidden planting exposed to sunlight, the geographic coordinates of which are 00°48′20.9″ S, 47°33′57.3″ W. Specimen (C) of *M. multiflora* was collected on 06/18/2019 in an area of secondary forest (capoeira) and the species of *E. florida* on 09/21/2019 in a lowland area on the banks of the Curral river. The exsiccate was incorporated in the collection of the João Murça Pires Herbarium (MG) of Museu Paraense Emílio Goeldi, in the collection of Aromatic Plants of the Amazon, Belém, Pará, and were registered under Voucher number MG231882 (*M. multiflora* A), MG231881 (*M. multiflora* B), MG231882 (*M. multiflora* C) and MG237472 (*E. florida*).

### 3.2. Preparation of Botanical Material

The leaves of *E. florida* and *M. multiflora* (A, B and C) were dried for 5 days at 35 °C in an oven with air circulation before being crushed in a knife mill. The moisture content was analyzed using an ID50 infrared humidity determiner in the temperature range of 60–180 °C, with a 1 °C increment and bidirectional RT-232 °C output.

### 3.3. Extraction of Essential Oils

The samples were subjected to hydrodistillation in modified Clevenger-type glass systems for 3 h, coupled to a refrigeration system to maintain the condensation water at around 12 °C. After the extraction, the oils were centrifuged for 5 min at 3000 rpm, dehydrated with anhydrous sodium sulfate and centrifuged again under the same conditions. Oil yield was calculated in mL/100 g. The oils were stored in amber glass ampoules, sealed with flame and stored in a refrigerator at 5 °C.

### 3.4. Chemical Composition Analysis

The chemical compositions of the EOs of *M. multiflora* (A, B and C) and *E. florida*, were analyzed using a Shimadzu QP-2010 plus (Kyoto, Japan), a gas chromatography system equipped with an Rtx-5MS capillary column (30 m × 0.25 mm; 0.25 µm film thickness) (Restek Corporation, Bellefonte, PA, USA) coupled to a mass spectrometer (GC/MS) (Shimadzu, Kyoto, Japan). The programmed temperature was maintained at 60–240 °C at a rate of 3 °C/min, with an injector temperature of 250 °C, helium as the carrier gas (linear velocity of 32 cm/s, measured at 100 °C) and a splitless injection (1 μL of a 2:1000 hexane solution), using the same operating conditions as described in the literature [62]. Except for the carrier hydrogen gas, the components were quantified using gas chromatography (CG) on a Shimadzu QP-2010 system (Kyoto, Japan), equipped with a flame ionization detector (FID) (Kyoto, Japan), under the same operating conditions as before. The retention index for all volatile constituents was calculated using a homologous series of n-alkanes (C8–C40), Sigma-Aldrich (San Luis, MO, USA), according to van den Dool and Kratz [63]. The components were identified by comparison (i) of the experimental mass spectra with those compiled in libraries (reference) and (ii) their retention indices to those found in the literature [38,39].

### 3.5. Antimicrobial Activity of the Essential Oils

#### 3.5.1. Microorganisms

To analyze the antifungal activity, 5 fungal species were used (*Candida albicans* INCQS-40175, *C. tropicalis* ATCC 6258, *C. famata* ATCC 62894, *C. krusei* ATCC 13803 and *C. auris* IEC-01). All isolates were cultivated in Sabouraud agar at 37 °C for 48 h before the beginning of the tests. These tests were performed at the Laboratory of Superficial and Systemic Mycoses of Instituto Evandro Chagas (IEC).

#### 3.5.2. Agar Disc Diffusion Test

The antifungal activity of the essential oils was assessed using the agar disc diffusion method [64] with modifications. The paper filter discs (6 mm) were impregnated with 20 μL of each extract. The suspensions of the test microorganisms were prepared with 0.45% saline solution (0.5 on the McFarland scale). Each microorganism suspension was spread on the surface of Sabouraud agar culture medium in Petri dishes (15 × 90 mm). Then, the paper discs impregnated with the extracts were placed on the surface of the plates inoculated with the microorganisms. The plates were incubated for 48 h at 37 °C. After this period, visual readings were taken, observing the presence of growth inhibition zone measured in millimeters, with the help of a millimeter ruler. As a positive control, paper discs were impregnated with nystatin solution. All tests were carried out in duplicate. Inhibition zones ≥8 mm indicated that the microorganism was sensitive to the tested essential oil, according to the classification proposed by the authors [65,66,67].

#### 3.5.3. Broth Microdilution: Determination of MIC

To determine the minimum inhibitory concentration (MIC), the oils from specimens A and C of *M. multiflora* were selected (they presented sufficient quantities to carry out all tests). The susceptibility of the microorganisms to the 5 extracts was determined by the broth microdilution method recommended by the *“US National Committee for Clinical Laboratory Standards”* (NCCLS), with adaptations. Five microorganisms were tested (*Candida albicans* INCQS-40175, *C. tropicalis* ATCC 6258, *C. famata* ATCC 62894, *C. krusei* ATCC 13803 and *C. auris* IEC-01). The microorganisms were cultivated in Sabouraud agar at 37 °C for 48 h. From these cultures, cellular suspensions similar to the McFarland scale 0.5 were prepared. In a 96-well plate, serial dilution at a ratio of 2 of each extract to be tested was performed, starting from 10% (20/180 μL), in a final volume of 100 μL. Then, 100 µL of the yeast suspension was added. The final concentration in each well reached 50% µL/mL, 25% µL/mL, 12.5 µL/mL, 6.2 µL/mL, 3.1 µL/mL, 1.5 µL/mL, 0.7 µL/mL, 0.3 µL/mL, 0.19 µL/mL and 0.0 µL/mL. After adding the yeast to the previously diluted oils, the plate was incubated in a bacteriological incubator at 37 °C for 48 h, and at the end, the readings were taken visually, and the lowest concentration of extract capable of inhibiting visible fungal growth was recorded. The test was carried out in duplicate [68].

#### 3.5.4. Broth Microdilution: Determination of MFC

After completion of the test and visual reading of the broth microdilution to determine MIC, the test to determine the minimum fungicidal concentration (MFC) was carried out. The test consisted of plating 10 μL of each dilution in Sabouraud agar and incubating them in a bacteriological incubator at 37 °C for 48 h. After this period, the lowest dilution capable of killing 99.5% of the original inocula was recorded. The test was carried out in duplicate [68].

### 3.6. Statistical Analysis

The EO composition data required hierarchical cluster analysis (HCA) and principal component analysis (PCA) to establish composition patterns between the species. The data were processed by Minitab Statistical Software 17 trial version (State College, PA, USA). The HCA was carried out by adopting the similarity using Euclidean distance through a 4 × 28 correlation matrix, and the PCA was carried out using the covariance matrix of the samples since all input data were presented as the mass percentage (≥2%). Both HCA and PCA were carried out using all the identified compounds as variables [36,69].

## 4. Conclusions

The chemical profile of the essential oils was strongly characterized by the sesquiterpene class, with α-bulnesene (26.79%), (*E*)-nerolidol (44.4%) and (*E*)-nerolidol (92.21%) in the essential oils of *Myrcia multiflora* (A, B and C), and selina-3,11-dien-6-α-ol (12.93%) in *Eugenia florida* as main constituents, and there was a significant difference between the chemical profiles of the studied species, which in a way is associated with different types of ecosystems and collection periods.

By chemometric analysis of hierarchical clustering analysis (HCA) and principal component analysis (PCA), it was possible to verify this expressive difference in the chemical composition of essential oils. In HCA and PCA there was the formation of three distinct groups of chemical profiles: Group I being formed by the sample *M. multiflora* (A) and Group II by the sample *E. florida*, both with a significant difference of approximately 96.08%. Group III was formed by the samples *M. multiflora* (B) and *M. multiflora* (C), with a similarity degree of 38.1%. This formation of groups is related to the presence of chemical compounds present in the essential oils of each studied species.

The essential oils of *Myrcia multiflora* showed high inhibition potential against the fungal species *C. albicans*, *C. famata* and *C. krusei*, which may be related to the hydrophobicity of the terpenoid class, mainly of the compounds α-bulnesene, pogostol, δ-amorphene and (*E*)-nerolidol. However, new studies have to be carried out to prove the antimicrobial potential of the studied species and their actual impact on the obstruction of fungal cells.

In view of the search for new natural pathogens, the fungicidal potential presented in *M. multiflora* essential oils may be promising for the development of natural agents that inhibit the action of diseases caused by these phytopathogens.

## Figures and Tables

**Table 2:** In vitro effect of the botanical extracts on medically important yeasts assessed by the agar disc diffusion method.

	Halo Diameter (mm)			
Fungal Species	*Myrcia multiflora* (A)	*Eugenia florida*	*M. multiflora* (B)	*M. multiflora* (C)
*C. albicans*	8	7	9	7
*C. tropicalis*	8	8	11	8
*C. krusei*	6	6	10	8
*C. famata*	9	8	10	8
*C. auris*	9	8	10	8

**Table 3:** Minimal inhibitory concentration (MIC) and minimal fungicidal concentration (MFC) of extracts on medically important yeasts; data expressed in µL/mL.

	*Myrcia multiflora* (A)		*M. multiflora* (C)	
Fungal Species	MIC	MFC	MIC	MFC
*C. albicans*	50	50	50	50
*C. tropicalis*	50	a	50	>50
*C. krusei*	6.25	>25	12.5	50
*C. famata*	0.78	3.12	12.5	>50
*C. auris*	3.12	>12.5	5	>50

A reduction in colonial growth was observed after plating.

## Data Availability

All supporting data can be obtained from the corresponding author upon formal request.

## Sample Availability

Samples of *Myrcia multiflora* and *Eugenia florida*. The essential oil of the Museu Paraense Emílio Goeldi is available from the authors.
